# Effect of Virtual Reality-Based Mindfulness Program vs Audio-Guided Mindfulness on Depression, Sleep, and Quality of Life in Dementia Family Caregivers: An Exploratory Randomized Controlled Trial

**DOI:** 10.7150/ijms.126415

**Published:** 2026-01-30

**Authors:** Dorothy Bai, Yu-Hua Wang, Yu-Fang Lin, Megan F. Liu

**Affiliations:** 1 School of Gerontology and Long-Term Care, College of Nursing, Taipei Medical University, Taipei City, Taiwan.; 2 Post-Baccalaureate Program in Nursing, Asia University, Taichung, Taiwan.; 3 College of Interdisciplinary Studies, Taipei Medical University, Taipei City, Taiwan.

**Keywords:** virtual reality, mindfulness, dementia, family caregivers, quality of life

## Abstract

**Background:**

Family caregivers of individuals with dementia often experience significant psychological and physical burdens. While mindfulness-based interventions have shown potential in improving caregiver well-being, the effects of integrating virtual reality (VR) technology into these interventions remain underexplored.

**Objective:**

This study compared a VR-based mindfulness program with an audio-guided mindfulness program on depression, sleep quality, and quality of life among family caregivers of people with dementia.

**Methods:**

An exploratory randomized controlled trial was conducted with 16 dementia family caregivers. Participants were randomly assigned to a VR-based mindfulness intervention group or an audio-guided mindfulness active control group. The six-week intervention included practices such as mindful breathing, body scanning, and yoga. The primary outcome was depression (Center for Epidemiologic Studies Depression Scale, CES-D). Secondary outcomes were sleep quality (Chinese Pittsburgh Sleep Quality Index, CPSQI) and quality of life (WHOQOL-BREF Taiwan version). Outcomes were assessed at baseline and post-intervention. Within-group changes were assessed using the Wilcoxon signed-rank test, and between-group differences in change scores were compared using the Mann-Whitney U test.

**Results:**

Both groups showed significant within-group improvements in depression (VR: -4.50, *p* = .012; audio: -4.00, *p* = .012), sleep quality (VR: -1.50, *p* = .012; audio: -1.50,* p* = .017), and quality of life (VR: +2.00, *p* = .012; audio: +3.00, *p* = .012). Although between-group differences were not statistically significant, no VR-related adverse symptoms were reported, and the findings support further evaluation of VR-delivered mindfulness in larger trials.

**Conclusions:**

Both VR-based and audio-guided mindfulness interventions were associated with within-group improvements in depression, sleep, and quality of life in dementia caregivers. VR offers an engaging alternative for mindfulness delivery, with potential to enhance user experience. Given the small sample size, these findings should be interpreted cautiously, and larger studies are needed to examine VR's added benefits.

## Introduction

Dementia is a progressive neurodegenerative condition characterized by cognitive decline, behavioral disturbances, and psychological symptoms 1-3. These challenges extend beyond the affected individuals and heavily impact family caregivers, who often assume primary responsibility for providing daily care 4-5. The caregiving role is frequently associated with significant psychological and physical burdens, including emotional distress, depression, poor sleep quality, and diminished quality of life 5-6. While numerous caregiver support programs have been developed to address these issues, many offer only temporary relief and fail to sufficiently mitigate the deeper and more persistent mental health challenges experienced by caregivers 7-8.

Mindfulness-based interventions (MBIs) have emerged as a promising approach to enhance psychological well-being in both clinical and non-clinical populations 9-10. Defined as the practice of intentionally focusing attention on the present moment without judgment, mindfulness has been associated with reductions in anxiety, depression, and stress, along with improvements in sleep quality and overall quality of life 11-12. For dementia caregivers, MBIs such as mindfulness-based stress reduction (MBSR) have demonstrated beneficial effects on emotional resilience, caregiving satisfaction, and mental health 13-15.

Recent advancements in digital health technologies have opened new possibilities for delivering psychological interventions 16. Among them, virtual reality (VR) has gained attention for its immersive and interactive capabilities, which simulate real-world environments and enhance user engagement 17. VR has shown promise in a variety of healthcare applications, including pain management, psychological therapy, and caregiver education 18-19. In dementia care, VR has been used to foster empathy among caregivers and provide simulated experiences to enhance understanding of the disease 20. VR-augmented mindfulness has also been explored as a method to facilitate relaxation and reduce stress in various settings 17, 21-22. Immersive VR may support mindfulness practice by increasing perceived presence and engagement, which may help users sustain attention and reduce distraction during guided sessions 22-23. Prior VR-mindfulness research suggests VR can be used as a delivery modality to facilitate mindfulness skills practice, although effects may vary by population, intervention design, and outcome measures 23.

Despite growing interest in VR and mindfulness independently, little is known about the combined effects of VR-integrated mindfulness interventions for family caregivers of people with dementia. This study aimed to investigate whether a VR-based mindfulness program could improve depression, sleep quality, and quality of life in dementia caregivers compared to a traditional audio-guided mindfulness program.

## Methods

### Study design and setting

This study employed an exploratory parallel randomized controlled trial (RCT) method to evaluate the effectiveness of a VR-based mindfulness intervention among family caregivers of individuals with dementia. This study adheres to the CONSORT guidelines for reporting randomized controlled trials. The trial was registered on ClinicalTrials.gov (Identifier: NCT06034249). The trial was registered retrospectively. Participants were recruited from various dementia care centers in Taiwan between August and November 2022.

### Participants

Eligible participants were family caregivers of individuals diagnosed with dementia. Inclusion criteria were: (1) being a family caregiver responsible for caring for a family member diagnosed with dementia by a medical institution, (2) having the ability to communicate in Mandarin or Taiwanese, and (3) providing informed consent by signing the consent form. Individuals with visual or auditory impairments were excluded. This study was designed as an exploratory randomized controlled trial to obtain preliminary estimates of feasibility and potential effects. A formal a priori power calculation was not performed. The target sample size was determined pragmatically based on recruitment feasibility within the study period, available resources for intervention delivery, and follow-up capacity. Therefore, the findings are intended to inform the design and sample size planning of a future adequately powered trial.

### Randomization and allocation concealment

**Figure 1:**
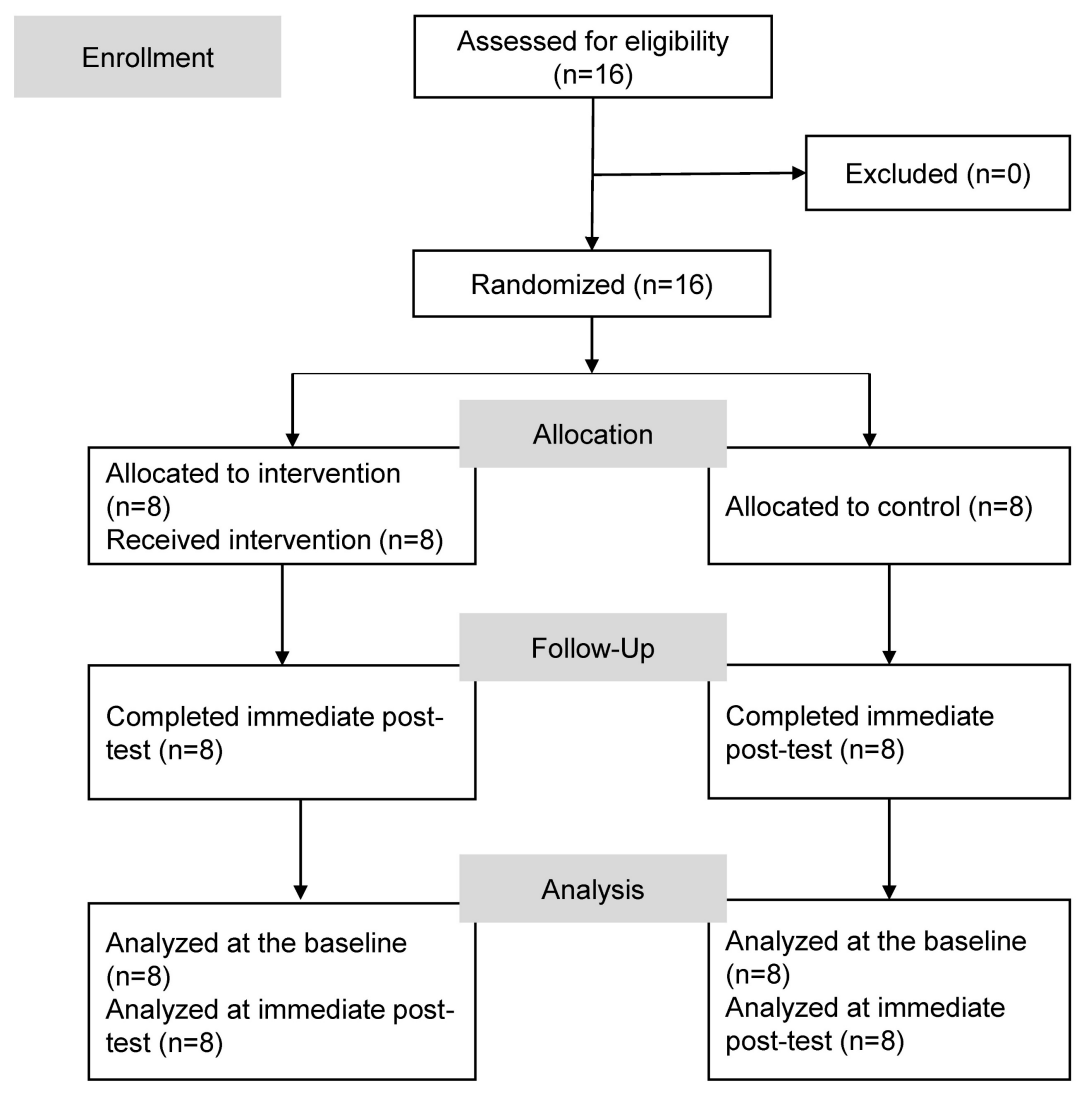
Flow diagram of the study.

Participants who consented to join the study were randomized using a computer-generated random number sequence, with 1 indicating assignment to the intervention group and 2 indicating assignment to the control group. The randomization sequence was generated by a researcher not involved in participant recruitment, intervention delivery, or outcome assessment. Allocation was concealed using sequentially numbered, opaque, sealed envelopes that were opened only after completion of baseline assessment and enrollment. Figure 1 shows the flow diagram of this study. In total, 16 participants were enrolled and were randomly assigned to either the intervention group or control group.

### Intervention

The intervention group underwent a 6-week VR-based mindfulness intervention encompassing practices such as breathing observation, body scanning, mindful yoga, mindful walking, holistic meditation, and non-selective awareness, with each session lasting 40-60 minutes (Table 1). The six-week mindfulness curriculum was adapted from commonly used mindfulness-based practices and was organized to progressively build skills across sessions. The weekly themes included breath awareness, body scan, mindful movement such as gentle yoga, walking meditation, and open monitoring style practices. The mindfulness guidance was standardized across sessions to support consistency of delivery.

In the VR group, the mindfulness guidance was delivered within an immersive VR experience designed to support attention regulation and engagement during practice. The VR application presented a stationary, low-motion virtual environment with minimal interaction demands, with no navigation or task requirements, allowing participants to focus on the guided mindfulness practice rather than on operating the VR system. Participants wore the head-mounted display for approximately 30 minutes within each 40-60-minute session, and it could be removed at any time upon request. The VR environment was selected to support sustained attention and engagement during the weekly mindfulness practice.

To address potential VR-related discomfort, participants received orientation and brief familiarization before the first session and were encouraged to pause or stop the VR experience at any time if they experienced dizziness, nausea, or discomfort. Sessions were conducted with staff available to monitor participants and provide support as needed, and any adverse symptoms were recorded. The six-week duration was chosen to balance feasibility for dementia family caregivers with sufficient time to establish regular mindfulness practice, consistent with prior caregiver-focused mindfulness intervention designs 24.

The control group received mindfulness-based audio files for six weeks. Participants were instructed to follow the audio guidance according to the weekly themes and practice schedule as shown in Table 1. This condition served as an active comparator, providing mindfulness content without VR delivery.

### Outcome measures

The primary outcome was change in depressive symptoms measured by the Center for Epidemiologic Studies Depression Scale (CES-D; score range 0-60), with higher scores indicating a higher frequency of depressive symptoms 25. Secondary outcomes included sleep quality assessed by the Chinese Pittsburgh Sleep Quality Index (CPSQI; score range 0-21), with higher scores indicating worse sleep quality 26, and quality of life measured by the Taiwan version of the World Health Organization Quality of Life Brief questionnaire (WHOQOL-BREF Taiwan version), with higher scores indicating better quality of life 27. Data were collected at baseline and post-intervention by trained researchers.

### Statistical analysis

Data were analyzed using PASW SPSS Statistics 28.0 software. Descriptive statistics included counts, percentages, medians, and interquartile ranges (IQRs). The Mann-Whitney U test was employed to analyze continuous variables, while a Chi-squared test or Fisher's exact test, as appropriate, was used for categorical variables. To assess within-group changes (pre- to post-intervention) in depression, sleep quality, and QOL, the Wilcoxon signed-rank test was applied. The Mann-Whitney U-test was used to compare changes between groups. To facilitate interpretation of the magnitude of change without additional standardized effect size metrics, we report group medians (IQRs) at baseline and the corresponding pre-to-post change scores (delta) for each outcome.

### Ethical considerations

The study protocol was reviewed and approved by the Joint Institutional Review Board of Taipei Medical University Hospital (N202207036). Informed consent was obtained from all participants prior to enrollment. This study was conducted in accordance with the principles of the Declaration of Helsinki.

## Results

Sixteen participants were enrolled in the study and analyzed. Of these, eight were assigned to the intervention group and the remaining eight to the control group. Table 2 shows demographic characteristics of caregivers and care recipients. Caregivers ranged in age from 39 to 81 years, with a median age of 56 in both groups. The majority were female. Average daily caregiving time differed between groups: VR 8.00 hours (IQR 9.75) vs audio-guided 20.00 hours (IQR 16.00). This difference was statistically significant *(p* = 0.045). Initial assessments of depressive symptoms, sleep quality, and QOL respectively using the CES-D, CPSQI, and WHOQOL-BREF revealed no significant differences between the two groups at the baseline. No VR-related adverse symptoms were reported during the intervention.

Care recipients ranged in age from 56 to 94 years. In the intervention group, the median age was 83.5 years (IQR 18.75), with a higher proportion of male patients. In the control group, the median age was 85.5 years (IQR 8.5), with a higher proportion of female patients. Educational backgrounds varied, with a higher percentage of the intervention group's care recipients having a college-level education or above, while most in the control group had a high school education or below. However, these differences were not statistically significant. The level of disability and dementia severity were comparable between the two groups.

Post-intervention, both groups exhibited a significant reduction in depressive symptoms (Table 3). The intervention group's median CES-D score decreased, indicating a notable improvement (*p* = .012), and a similar trend was observed in the control group (*p* = .012). The intervention group showed a significant decrease in CPSQI scores (*p* = .012), reflecting better sleep patterns post-intervention. The control group mirrored those results with a notable improvement in their sleep quality (*p* = .017). Participants in both groups reported an enhanced QOL following the intervention. Significant improvements were observed across all domains and total scores of the WHOQOL-BREF in both the intervention (*p* values ranging .011 to .016) and control groups.

Despite the significant within-group improvements, the comparative analysis results between the intervention and control groups in Table 4 revealed no statistically significant differences in changes in depression scores, sleep quality, or overall QOL following the intervention (*p*>.05 for all comparisons). This finding suggests that while mindfulness interventions are beneficial, the addition of VR did not result in a statistically significant enhancement of these benefits compared to traditional mindfulness practices alone.

## Discussion

This study evaluated the effects of a virtual reality-based mindfulness intervention on depression, sleep quality, and quality of life among family caregivers of individuals with dementia. Both the VR-based intervention and the audio-guided mindfulness group showed significant within-group improvements across outcomes while between-group differences were not statistically significant. Given the small sample size, these findings should be interpreted cautiously and considered preliminary evidence to inform future studies with larger caregiver samples. Because both groups received the same mindfulness content and the audio-guided condition served as an active comparator, the between-group comparison assessed the incremental value of VR delivery beyond mindfulness practice itself. In this six-week exploratory randomized controlled trial, VR did not provide a detectable additional benefit on depression, sleep quality, or quality of life beyond mindfulness practice.

The reduction in depressive symptoms observed in both groups is noteworthy, particularly given the moderate baseline levels of depression among participants. Depression is highly prevalent among dementia caregivers and is frequently associated with prolonged emotional stress, behavioral challenges in care recipients, and a lack of sufficient social or institutional support 5, 28-29. The significant reduction in CES-D scores across both groups supports previous research highlighting the effectiveness of mindfulness-based interventions in alleviating caregiver depression 13. Importantly, these results suggest that even audio-guided mindfulness programs, which are more accessible and less resource-intensive, can offer meaningful psychological benefits. Taken together with the null between-group findings, these results further highlight mindfulness practice itself as the key active ingredient, regardless of whether it is delivered via VR or audio guidance.

Sleep quality also improved significantly in both groups. Caregivers in this study reported poor sleep at baseline, a finding that echoes existing literature attributing caregiver sleep disturbance to psychological distress, caregiving intensity, and nighttime disruptions by individuals with dementia 30-31. Although immersive VR could theoretically enhance user engagement and deepen relaxation, no superior improvements in sleep were noted in the VR group compared to the control. This pattern suggests that mindfulness-related skills (eg, attentional regulation and reduced rumination) may be the primary drivers of sleep improvement, with delivery format (VR vs audio) playing a secondary role over six weeks.

Enhancements in caregiver QOL were evident across all WHOQOL-BREF domains. While the VR group experienced slightly greater gains, these differences were not statistically significant. Nonetheless, the positive changes observed in both groups reinforce the value of mindfulness for promoting psychological resilience and enhancing caregivers' perceived well-being 7, 32. Given the growing demand for non-pharmacological support options, mindfulness, delivered in either digital or traditional formats, offers a promising tool for caregiver support. This again supports the interpretation that mindfulness practice itself, rather than the delivery modality, likely accounts for the observed improvements in quality of life.

This study has several limitations. The small sample size limited statistical power and generalizability; therefore, findings should be interpreted cautiously and considered preliminary evidence to inform future studies with larger samples. Participants covered a wide age range, and age-related differences in familiarity with digital technologies may influence engagement and acceptability of VR-based interventions. This exploratory trial was not powered to examine age-related moderation or to test mediation effects. Although randomization was implemented, baseline differences in average daily caregiving time may have introduced residual confounding and influenced outcome trajectories, particularly for depressive symptoms and sleep quality 33-34. Outcomes were assessed using self-reported questionnaires, which may be subject to reporting bias and may not capture objective changes in sleep or stress-related physiology. The six-week duration and limited follow-up may also have constrained detection of longer-term changes or incremental effects attributable to delivery format. In addition, VR-based delivery may introduce novelty or engagement effects, particularly early in use, which could influence self-reported outcomes independently of mindfulness skill acquisition. Finally, we did not include direct measures of attention, focus, or presence/immersion during mindfulness practice, limiting our ability to evaluate whether delivery format influenced concentration during sessions.

Future research should recruit larger and more diverse caregiver samples, and consider stratified randomization by caregiving load to reduce baseline imbalance. Longer intervention periods and/or follow-up may be needed to capture longer-term changes and potential incremental effects attributable to delivery format. Incorporating objective outcome measures such as biomarkers, sleep tracking, or physiological indicators would further strengthen study rigor. In addition, future studies should evaluate technology acceptance using a framework such as the Technology Acceptance Model, assess mindfulness skills using validated questionnaires such as the Mindful Attention Awareness Scale and the Five Facet Mindfulness Questionnaire, and include measures of presence/immersion or attention-related constructs to test whether VR (vs audio) better supports focused mindfulness practice and to examine how these factors relate to adherence and clinical outcomes.

## Conclusions

This exploratory randomized controlled trial found that both virtual reality-based and audio-guided mindfulness interventions were associated with significant within-group improvements in depression, sleep quality, and quality of life among family caregivers of people with dementia. However, compared with an active, audio-guided mindfulness program, the VR-based delivery did not demonstrate statistically greater benefits, suggesting no detectable incremental advantage of VR beyond mindfulness content in this six-week exploratory randomized controlled trial. Overall, the findings support mindfulness as a feasible and accessible strategy to promote caregiver well-being. Future studies with larger and more diverse samples, longer follow-up, and objective outcome measures are warranted to further evaluate whether VR delivery offers additional benefits and to clarify potential mechanisms.

## Figures and Tables

**Table 1:** Intervention Design and Content

	Intervention group (VR-based mindfulness intervention)	Control group (Audio-guided mindfulness program)
Week 1	Familiarization with the VR tool, mindful breathing, and meditation practice	Mindful breathing and meditation practice
Week 2	Body scan practice and mindful breathing	Body scan practice and mindful breathing
Week 3	Mindfulness meditation and recumbent mindfulness yoga	Mindfulness meditation and recumbent mindfulness yoga
Week 4	Mindful thought awareness practice and meditation practice	Mindful thought awareness practice and meditation practice
Week 5	Mindful standing yoga and breathing awareness	Mindful standing yoga and breathing awareness
Week 6	Thought awareness, labeling practice, and mindfulness meditation	Thought awareness, labeling practice, and mindfulness meditation

**Table 2:** Demographic Characteristics of Participants

Variables	Intervention group (*n*=8)	Control group (*n*=8)	*p*
	Count (Percentage)/Median (IQR)	Count (Percentage)/Median (IQR)	
Caregivers			
Gender			.467
Male	2 (25.0%)	0 (0.0%)	
Female	6 (75.0%)	8 (100.0%)	
Age (years)	56.00 (22.50)	56.00 (13.00)	.584
Educational level			1.000
High school and below	1 (12.5%)	2 (25.0%)	
College and above	7 (87.5%)	6 (75.0%)	
Employment status			.282
Full-time	4 (50.0%)	1 (12.5%)	
Retired/other	4 (50.0%)	6 (75.0%)	
Marital status			.315
Married	5 (62.5%)	2 (25.0%)	
Single/divorced/widowed	3 (37.5%)	6 (75.0%)	
Average daily caregiving time (hours)	8.00 (9.75)	20.00 (16.00)	.045*
Caregiving duration (years)	3.50 (6.00)	3.50 (3.38)	.757
Relationship to the care recipient			.282
Spouse	4 (50.0%)	1 (12.5%)	
Child/Grandchild	4 (50.0%)	7 (87.5%)	
Baseline data			
CES-D total score	16.00 (8.00)	15.00 (15.00)	.698
CPSQI total score	5.50 (4.25)	7.00 (7.25)	.624
WHOQOL-BREF			
Overall quality of life	3.00 (0.75)	3.00 (1.50)	.559
General aspect	3.00 (0.00)	3.00 (1.75)	.727
Physical domain	25.50 (3.50)	24.50 (9.50)	.784
Psychological domain	19.00 (3.25)	19.00 (7.25)	.524
Social relationships	14.00 (2.50)	13.00 (3.75)	.402
Environment	33.50 (4.50)	33.00 (7.75)	.716
Total score	99.00 (10.75)	95.50 (32.75)	.588
Care recipients			
Gender			1.000
Male	4 (50.0%)	3 (37.5%)	
Female	4 (50.0%)	5 (62.5%)	
Age (years)	83.50 (18.75)	85.50 (8.50)	.130
Educational level			.132
High school and below	2 (25.0%)	6 (75.0%)	
College and above	6 (75.0%)	2 (25.0%)	
Level of disability			.053
None	0 (0.0%)	3 (37.5%)	
Mild	6 (75.0%)	2 (25.0%)	
Moderate/severe	2 (25.0%)	3 (37.5%)	
Level of dementia			.836
Mild	2 (25.0%)	3 (37.5%)	
Moderate	4 (50.0%)	3 (37.5%)	
Severe	1 (12.5%)	2 (25.0%)	

*Note*: CES-D, Center for Epidemiologic Studies Depression Scale; CPSQI, Chinese Pittsburgh Sleep Quality Index; WHOQOL-BREF, Taiwan's World Health Organization Quality of Life Questionnaire Brief Version; IQR, interquartile range = third quartile (Q3) - first quartile (Q1).*p < .05

**Table 3:** Within-group Comparison of the Intervention and Control Groups after the Mindfulness Intervention

	Intervention group (*n*=8)			Control group (*n*=8)		
Variables	Pretest	Delta between pre and post scores	*p* _within group_	Pretest	Delta between pre and post scores	*p* _within group_
	Median (IQR)	Median (IQR)		Median (IQR)	Median (IQR)	
CES-D Total Score	16.00 (8.00)	-4.50 (10.50)	.012*	15.00 (15.00)	-4.00 (9.25)	.012*
CPSQI Total Score	5.50 (4.25)	-1.50 (5.00)	.012*	7.00 (7.25)	-1.50 (2.50)	.017*
WHOQOL-BREF						
Overall quality of life	3.00 (0.75)	0.50 (1.00)	.011*	3.00 (1.50)	0.00 (0.75)	.016*
General aspect	3.00 (0.00)	0.00 (1.00)	.016*	3.00 (1.75)	0.00 (1.00)	.011*
Physical domain	25.50 (3.50)	2.00 (4.50)	.011*	24.50 (9.50)	1.00 (5.75)	.012*
Psychological domain	19.00 (3.25)	0.50 (3.75)	.011*	19.00 (7.25)	1.50 (2.75)	.012*
Social Relationships	14.00 (2.50)	0.00 (4.25)	.012*	13.00 (3.75)	0.50 (1.75)	.012*
Environment	33.50 (5.50)	1.50 (5.00)	.012*	33.00 (7.75)	1.50 (3.50)	.012*
Total Score	99.00(10.75)	2.00 (19.00)	.012*	95.50 (32.75)	3.00 (16.00)	.012*

*Note*: CES-D, Center for Epidemiologic Studies Depression Scale; CPSQI, Chinese Pittsburgh Sleep Quality Index; WHOQOL-BREF, Taiwan's World Health Organization Quality-of -Life Questionnaire Brief Version; IQR, interquartile range = third quartile (Q3) - first quartile (Q1).*p<.05.

**Table 4:** Comparisons between the Intervention Group and Control Group after the Mindfulness Intervention

	Pretest			Delta between pre and post scores		
Variables	Intervention group (*n*=8) Median (IQR)	Control group (*n*=8) Median (IQR)	*p*_between groups_	Intervention group (*n*=8) Median (IQR)	Control group (*n*=8) Median (IQR)	*p*_between groups_
CES-D Total score	16.00 (8.00)	15.00 (15.00)	.916	-4.50 (10.50)	-4.00 (9.25)	.958
CPSQI Total score	5.50 (4.25)	7.00 (7.25)	.668	-1.50 (5.00)	-1.50 (2.50)	.790
WHOQOL-BREF						
Overall quality of life	3.00 (0.75)	3.00 (1.50)	.682	0.50 (1.00)	0.00 (0.75)	.550
General aspect	3.00 (0.00)	3.00 (1.75)	.682	0.00 (1.00)	0.00 (1.00)	.906
Physical domain	25.50 (3.50)	24.50 (9.50)	.598	2.00 (4.50)	1.00 (5.75)	.524
Psychological domain	19.00 (3.25)	19.00 (7.25)	.672	0.50 (3.75)	1.50 (2.75)	.454
Social relationships	14.00 (2.50)	13.00 (3.75)	.486	0.00 (4.25)	0.50 (1.75)	.708
Environment	33.50 (5.50)	33.00 (7.75)	.634	1.50 (5.00)	1.50 (3.50)	.457
Total score	99.00 (10.75)	95.50 (32.75)	.636	2.00 (19.00)	3.00 (16.00)	.916

*Note*: CES-D, Center for Epidemiologic Studies Depression Scale; CPSQI, Chinese Pittsburgh Sleep Quality Index; WHOQOL-BREF, Taiwan's World Health Organization Quality-of -Life Questionnaire Brief Version; IQR, interquartile range = third quartile (Q3) - first quartile (Q1).*p <.05

## Data Availability

The datasets generated and/or analyzed during the current study are not publicly available due to institutional policies but are available from the corresponding author on reasonable request.
